# Critical analysis of the electroconvulsive therapy unit of centro hospitalar lisboa norte

**DOI:** 10.1192/j.eurpsy.2021.416

**Published:** 2021-08-13

**Authors:** C. Perestrelo Da Silva, A.C. Cordeiro, I. Chendo

**Affiliations:** Psiquiatria, Centro Hospitalar Universitário Lisboa Norte, Lisboa, Portugal

**Keywords:** Electroconvulsive therapy, schizophrénia, ECT, Major Depression

## Abstract

**Introduction:**

Electroconvulsivotherapy (ECT) one of the oldest treatments in biological psychiatry, is used nowaday mainly due to safety, efficacy and tolerability. Can be first-line treatment for mood disorders with catatonic or psychotic symptoms, and a second-line tretament to pharmacoherapy resistance or intolerable side effects.

**Objectives:**

To analyze the number of ECTs done, the number of patients submitted to this procedure in the ECT Unit in Centro Hospitalar Lisboa Norte (CHULN) comparing their diagnosis. To evaluate the number of patients that underwent maintenance and/or continuation treatment.

**Methods:**

Retrospective study involving patients submitted to ECT from 1 of January to 31 of December of 2019. A literature review exploring the use of ECT in psychiatry was conducted.

**Results:**

During the 12-month period were performed 179 sessions, corresponding to 18 patients. The diagnosis were schizophrenia, 55%, bipolar disorder, 39% and 6% with major depression. Only 28% underwent continuation and/or maintenance treatment.

**Conclusions:**

In this sample, of those diagnosed with schizophrenia, 90% were submitted to ECT due to oral therapy failure and 10% due to catatonia. Of those diagnosed with bipolar disorder 42.9% had a depressive episode and of these 14.2% had psychotic symptoms. This Unit is integrated in the biggest hospital of Portugal, it is import to understand the small number of patients submitted to this treatment and identify factors that may be preventing the referral of patients to this treatment. Clinicians may have the impression that ECT should be left as a last resort treatment which may explain the low percentage of major depression among our patients.

**Disclosure:**

No significant relationships.

